# Supplementary material for: Structure-Based Virtual Screening and De Novo Design to Identify Submicromolar Inhibitors of G2019S Mutant of Leucine-Rich Repeat Kinase 2
Source: Int J Mol Sci. 2022 Oct 24;23(21):12825. doi: 10.3390/ijms232112825 (PMC9654793; doi:10.3390/ijms232112825)
Supplement: Supplementary file 1 [file ijms-23-12825-s001.zip › ijms-1967696-supplementary.pdf]

International Journal of *Molecular Sciences*

MDPI

Supplementary Materials

Structure-Based Virtual Screening and De Novo Design to Identify Submicromolar Inhibitors of G2019S mutant of Leucine-Rich Repeat Kinase 2

Hwangseo Park, Taeho Kim, Kewon Kim, Ahyoung Jang, and Sungwoo Hong**

Department of Bioscience and Biotechnology, Sejong University, 209 Neungdong-ro, Kwangjin-gu, Seoul 05006, Korea
Center for Catalytic Hydrocarbon Functionalizations, Institute for Basic Science (IBS), Daejeon 34141, Korea
Department of Chemistry, Korea Advanced Institute of Science and Technology (KAIST), Daejeon 34141, Korea

Dose-response curve fits of **1-18** and **2a-2p** Figure S1

Figure S1. Dose-response curve fits of **1-18** and **2a-2p**, which were used to obtain their IC_{50} values with respect to (a) wild type and (b) G2019S mutant of LRRK2.

(a)

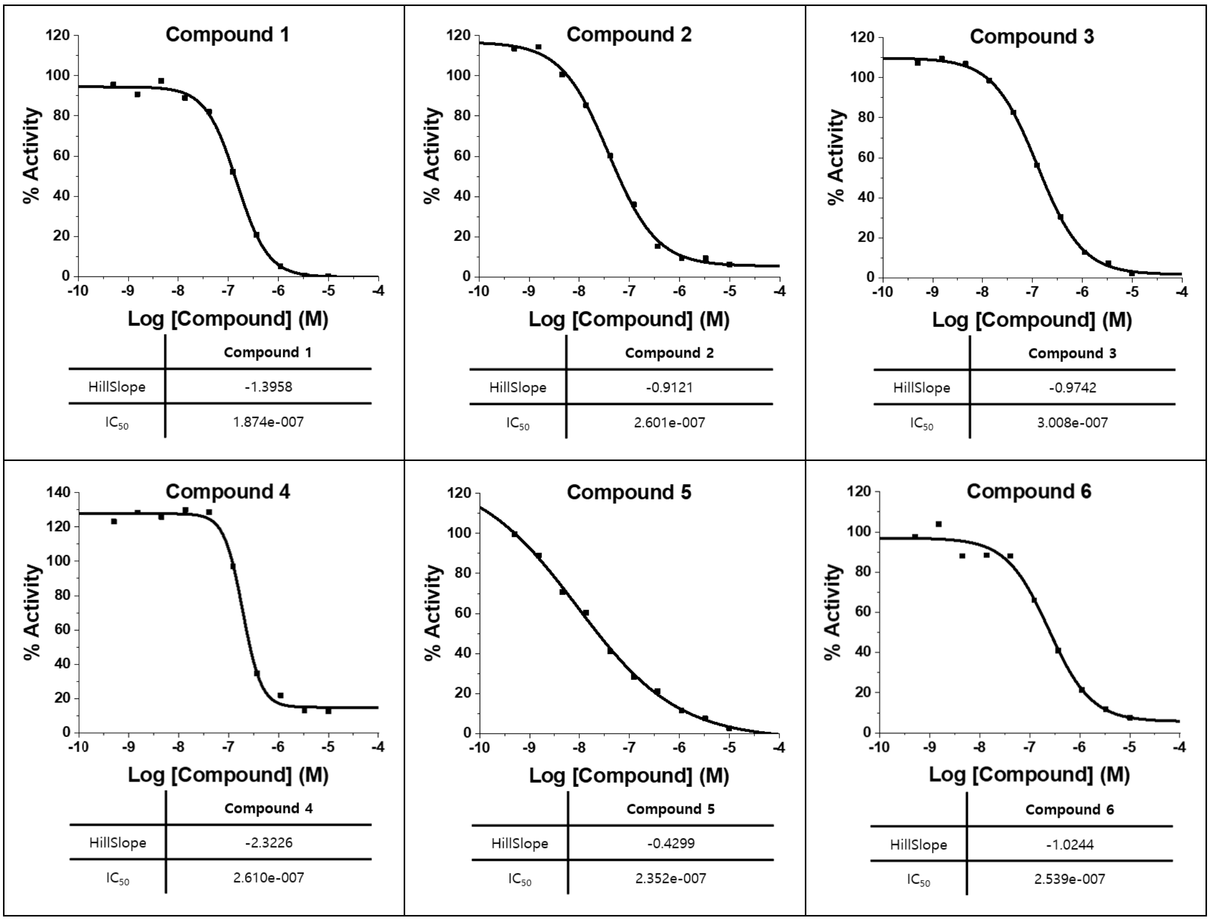
Compound 1
% Activity
Log [Compound] (M)
Compound 1
HillSlope -1.3958
IC50 1.874e-007
Compound 2
% Activity
Log [Compound] (M)
Compound 2
HillSlope -0.9121
IC50 2.601e-007
Compound 3
% Activity
Log [Compound] (M)
Compound 3
HillSlope -0.9742
IC50 3.008e-007
Compound 4
% Activity
Log [Compound] (M)
Compound 4
HillSlope -2.3226
IC50 2.610e-007
Compound 5
% Activity
Log [Compound] (M)
Compound 5
HillSlope -0.4299
IC50 2.352e-007
Compound 6
% Activity
Log [Compound] (M)
Compound 6
HillSlope -1.0244
IC50 2.539e-007

(b)

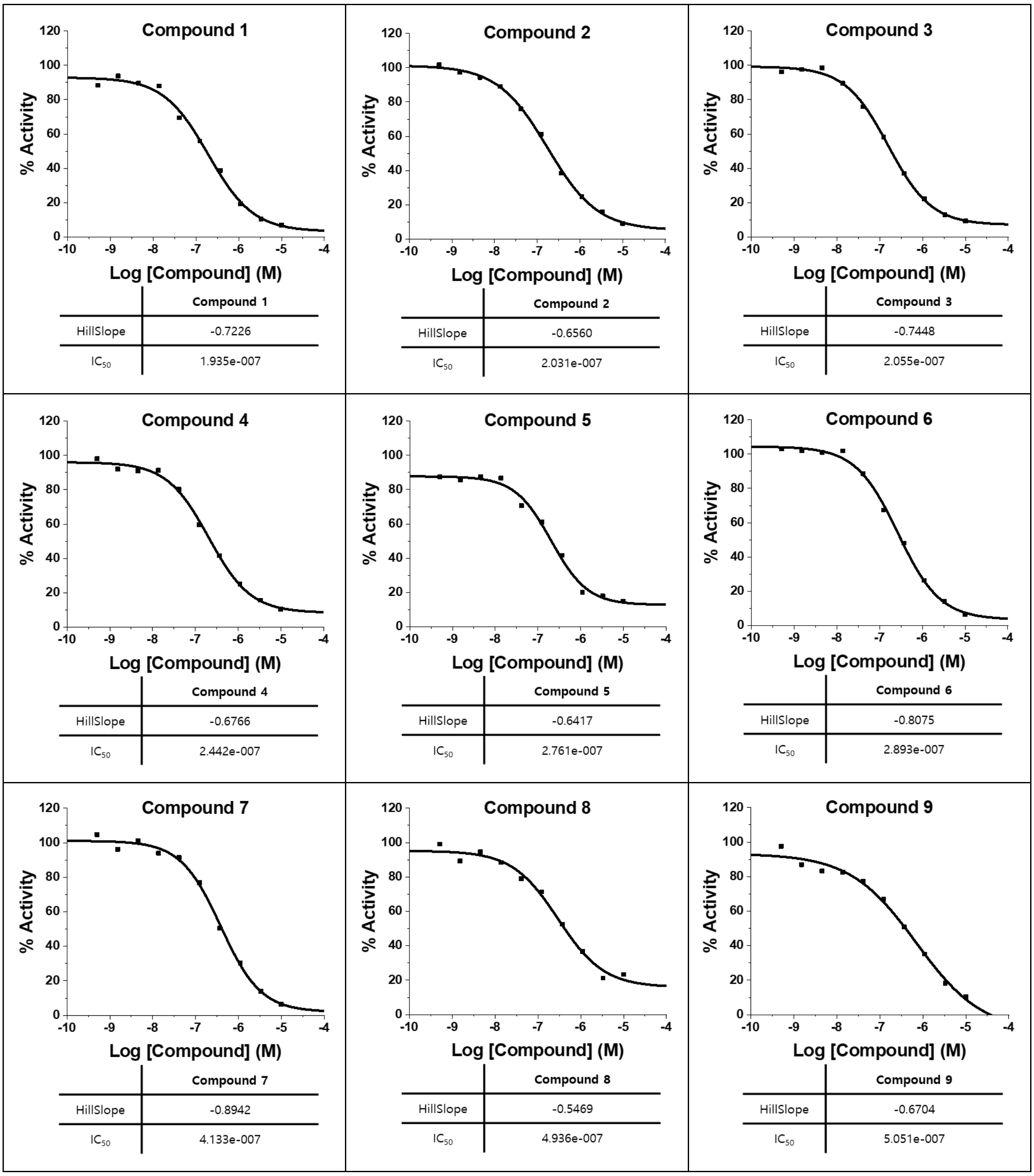
Compound 1
% Activity
Log [Compound] (M)
Compound 1
HillSlope -0.7226
IC50 1.935e-007
Compound 2
% Activity
Log [Compound] (M)
Compound 2
HillSlope -0.6560
IC50 2.031e-007
Compound 3
% Activity
Log [Compound] (M)
Compound 3
HillSlope -0.7448
IC50 2.055e-007
Compound 4
% Activity
Log [Compound] (M)
Compound 4
HillSlope -0.6766
IC50 2.442e-007
Compound 5
% Activity
Log [Compound] (M)
Compound 5
HillSlope -0.6417
IC50 2.761e-007
Compound 6
% Activity
Log [Compound] (M)
Compound 6
HillSlope -0.8075
IC50 2.893e-007
Compound 7
% Activity
Log [Compound] (M)
Compound 7
HillSlope -0.8942
IC50 4.133e-007
Compound 8
% Activity
Log [Compound] (M)
Compound 8
HillSlope -0.5469
IC50 4.936e-007
Compound 9
% Activity
Log [Compound] (M)
Compound 9
HillSlope -0.6704
IC50 5.051e-007

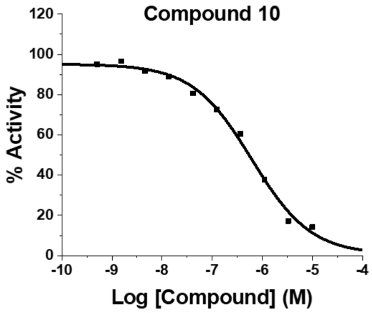

	Compound 10
HillSlope	-0.6771
IC_{50}	5.625e-007

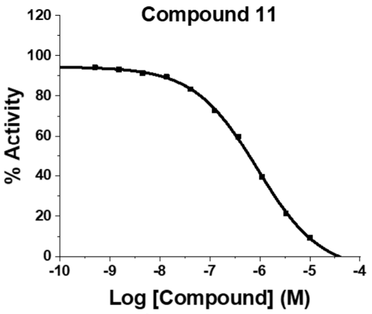

	Compound 11
HillSlope	-0.7297
IC_{50}	6.520e-007

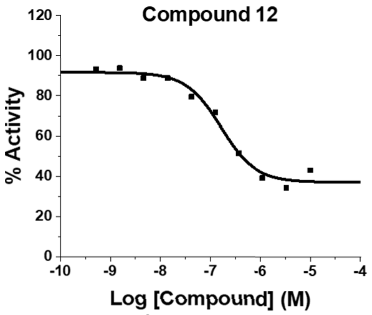

	Compound 12
HillSlope	-0.3386
IC_{50}	6.810e-007

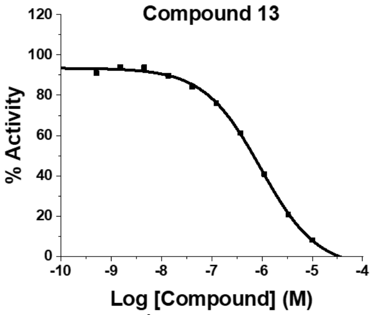

	Compound 13
HillSlope	-0.8409
IC_{50}	7.760e-007

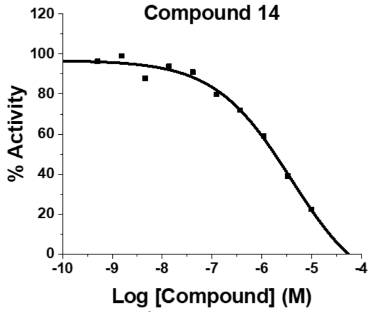

	Compound 14
HillSlope	-0.6752
IC_{50}	1.918e-006

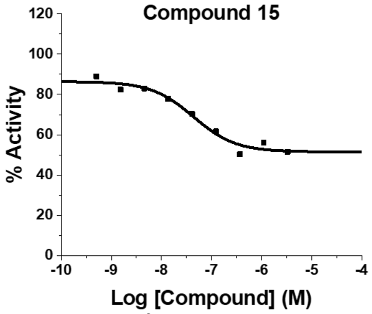

	Compound 15
HillSlope	-0.2248
IC_{50}	1.971e-006

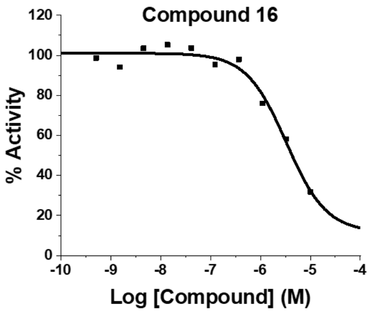

	Compound 16
HillSlope	-0.9755
IC_{50}	4.408e-006

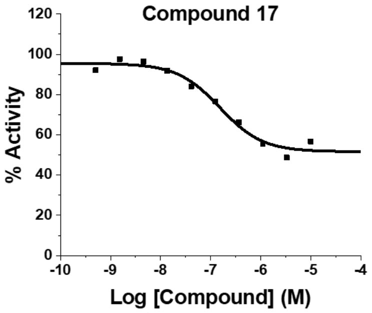

	Compound 17
HillSlope	-0.2857
IC_{50}	4.464e-006

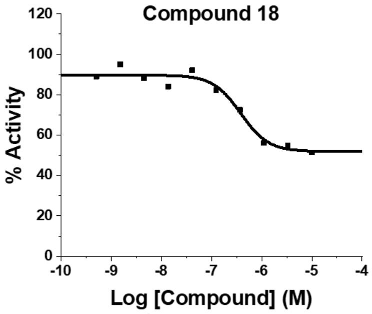

	Compound 18
HillSlope	-0.3816
IC_{50}	8.942e-006

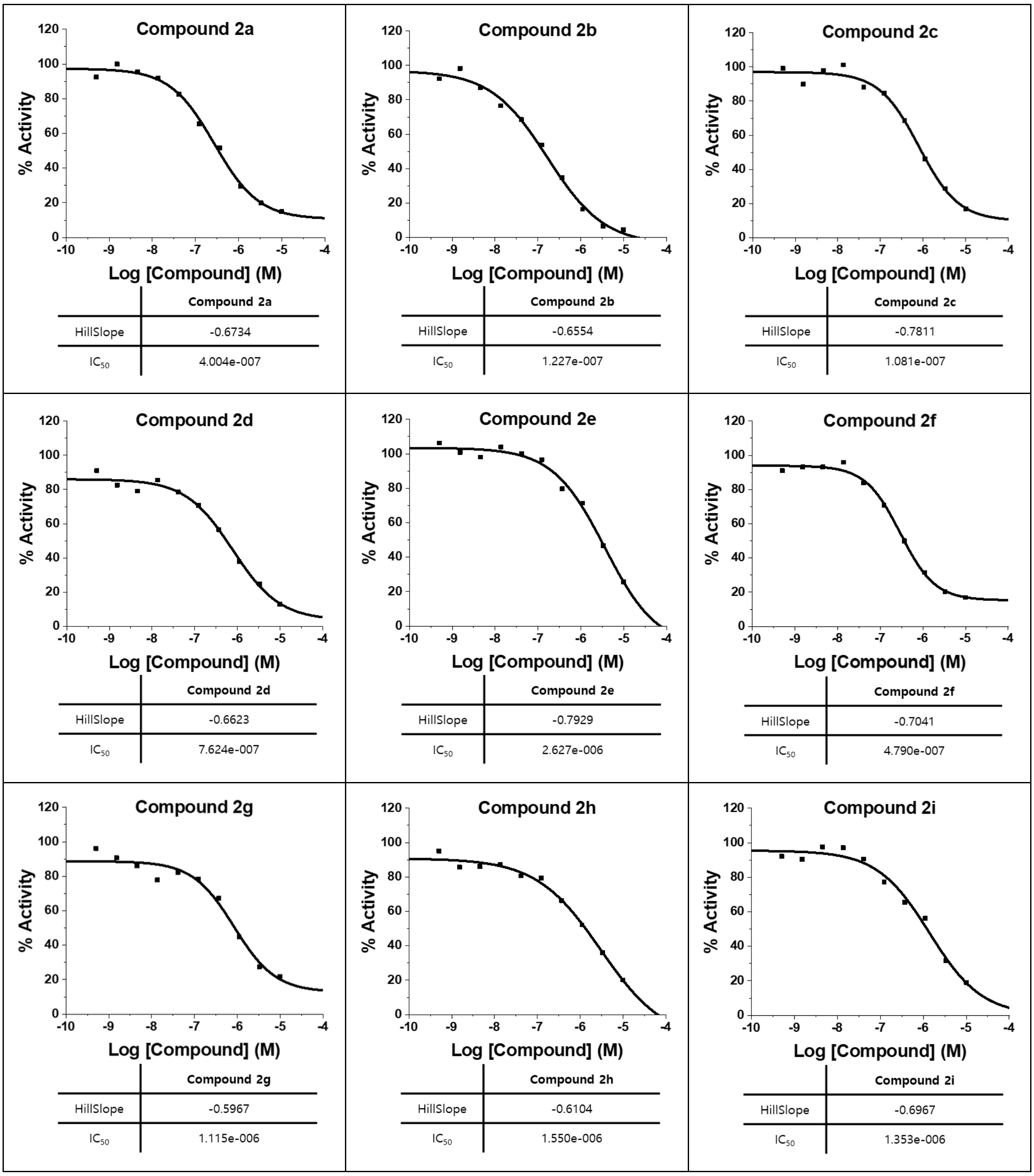
Compound 2a
% Activity
Log [Compound] (M)
HillSlope -0.6734
IC50 4.004e-007
Compound 2b
HillSlope -0.6554
IC50 1.227e-007
Compound 2c
HillSlope -0.7811
IC50 1.081e-007
Compound 2d
HillSlope -0.6623
IC50 7.624e-007
Compound 2e
HillSlope -0.7929
IC50 2.627e-006
Compound 2f
HillSlope -0.7041
IC50 4.790e-007
Compound 2g
HillSlope -0.5967
IC50 1.115e-006
Compound 2h
HillSlope -0.6104
IC50 1.550e-006
Compound 2i
HillSlope -0.6967
IC50 1.353e-006

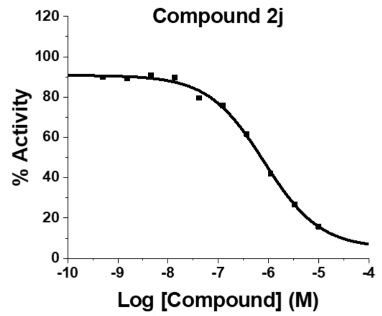

	Compound 2j
HillSlope	-0.6661
IC_{50}	8.830e-007

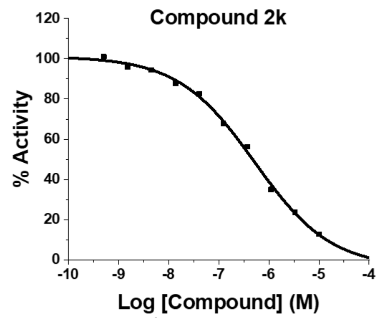

	Compound 2k
HillSlope	-0.5945
IC_{50}	4.379e-007

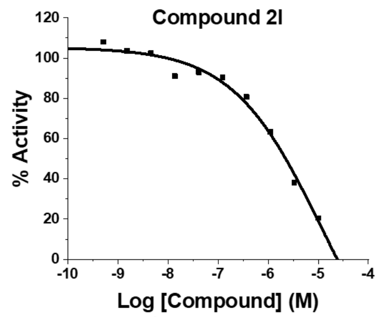

	Compound 2l
HillSlope	-0.7573
IC_{50}	1.823e-006

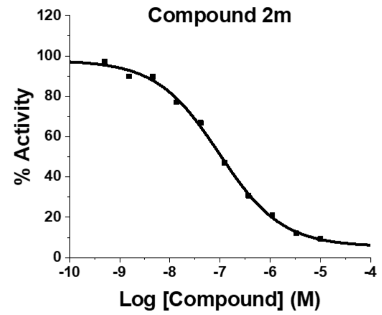

	Compound 2m
HillSlope	-0.6190
IC_{50}	1.218e-007

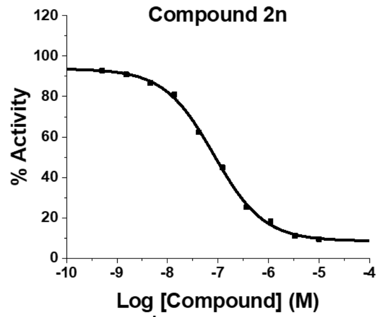

	Compound 2n
HillSlope	-0.6139
IC_{50}	8.937e-008

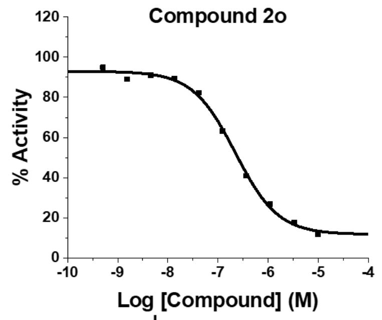

	Compound 2o
HillSlope	-0.7113
IC_{50}	3.159e-007

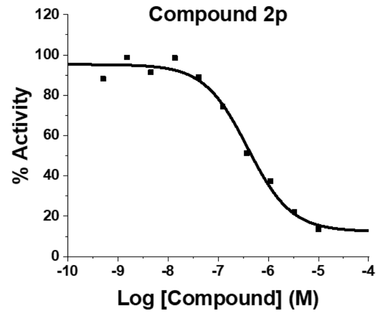

	Compound 2p
HillSlope	-0.7724
IC_{50}	6.125e-007
